# Intraoperative cangrelor during mechanical thrombectomy: is the systematic addition of aspirin needed?

**DOI:** 10.1093/esj/aakag124

**Published:** 2026-10-19

**Authors:** Małgorzata Milnerowicz, Federico Cagnazzo, Jean-Philippe Desilles, Maéva Kyheng, Julien Labreuche, Arturo Consoli, Benjamin Gory, Victor Dumas, Jildaz Caroff, Louis Veunac, Frederic Clarençon, Jean-Christophe Gentric, Christophe Cognard, Aymeric Rouchaud, Sebastien Soize, Bertrand Lapergue, Gaultier Marnat

**Affiliations:** Neuroradiology Department, Bordeaux University Hospital, Bordeaux, France; Neuroradiology Department, Montpellier University Hospital, Montpellier, France; Interventional Neuroradiology Department, Rothschild Foundation Hospital, Paris, France; Biostatistics Department, Lille University Hospital, Lille, France; Biostatistics Department, Lille University Hospital, Lille, France; Diagnostic and Interventional Neuroradiology Department, Foch Hospital, Suresnes, France; Neuroradiology Department, Nancy University Hospital, Nancy, France; Radiology Department, Poitiers University Hospital, Poitiers, France; Department of Interventional Neuroradiology, NEURI Brain Vascular Center, Bicêtre Hospital, APHP, Kremlin-Bicêtre, France; Radiology Department, Bayonne Hospital Center, Bayonne, France; Neuroradiology Department, Pitié-Salpétrière University Hospital, APHP, Paris, France; Neuroradiology Department, Brest University Hospital, Brest, France; Neuroradiology Department, Toulouse University Hospital, Toulouse, France; Neuroradiology Department, Limoges University Hospital, Limoges, France; Neuroradiology Department, Reims University Hospital, Reims, France; Neurology Department, Foch Hospital, Suresnes, France; Neuroradiology Department, Bordeaux University Hospital, Bordeaux, France

**Keywords:** antiplatelet therapy, ischemic stroke, rescue therapy, stenting, thrombectomy

## Abstract

**Objectives:**

In the hyperacute setting of mechanical thrombectomy (MT) for large-vessel-occlusion stroke, periprocedural platelet inhibition may be required in cases of refractory occlusions or underlying cervical or intracranial arterial disease at risk of reocclusion. Cangrelor is increasingly used in this context, although its use remains largely supported by observational evidence, and the impact of concomitant aspirin remains uncertain. This study investigated the safety and efficacy of the addition of intravenous aspirin to cangrelor during MT.

**Methods:**

We conducted a retrospective analysis of the Endovascular Treatment in Ischemic Stroke (ETIS) Registry across 34 French comprehensive stroke centres. All consecutive MT patients receiving intraoperative cangrelor between July 2018 and April 2025 were included, irrespective of the indication for intravenous antiplatelet therapy. The primary endpoint was 90-day favourable outcome (modified Rankin Scale [mRS] 0–2 or equal to pre-stroke mRS). Secondary outcomes included excellent outcome, early neurological improvement, angiographic recanalisation, day-1 arterial patency and intracranial haemorrhage (ICH). Propensity-score overlap weighting was used to adjust for confounding.

**Results:**

A total of 644 patients were analysed (cangrelor + aspirin combination: 226; cangrelor alone: 418). Favourable outcomes at 90 days were similar between groups (57.1% vs 58.8%; adjusted odds ratio [aOR] = 0.93; 95% confidence interval, 0.67–1.30; *P* = 0.69), as were excellent outcomes and early neurological improvement. Rates of successful and complete reperfusion were similar across groups. No differences were observed for symptomatic ICH (6.2% vs 9.2%; aOR = 0.66), any ICH, intra- and extracranial arterial patency on day-1 imaging, or 90-day mortality (22.3% vs 23.9%; aOR = 0.91).

**Conclusions:**

No significant differences in clinical, angiographic or safety outcomes were observed between cangrelor monotherapy and cangrelor combined with intravenous aspirin during MT. These findings provide comparative evidence on two cangrelor-based strategies currently used in clinical practice, although they should not be interpreted as demonstrating equivalence or excluding a potential benefit of adjunctive aspirin. Further prospective studies are needed to clarify the role of aspirin coadministration in this setting.

## Graphical abstract

**Graphical Abstract:**
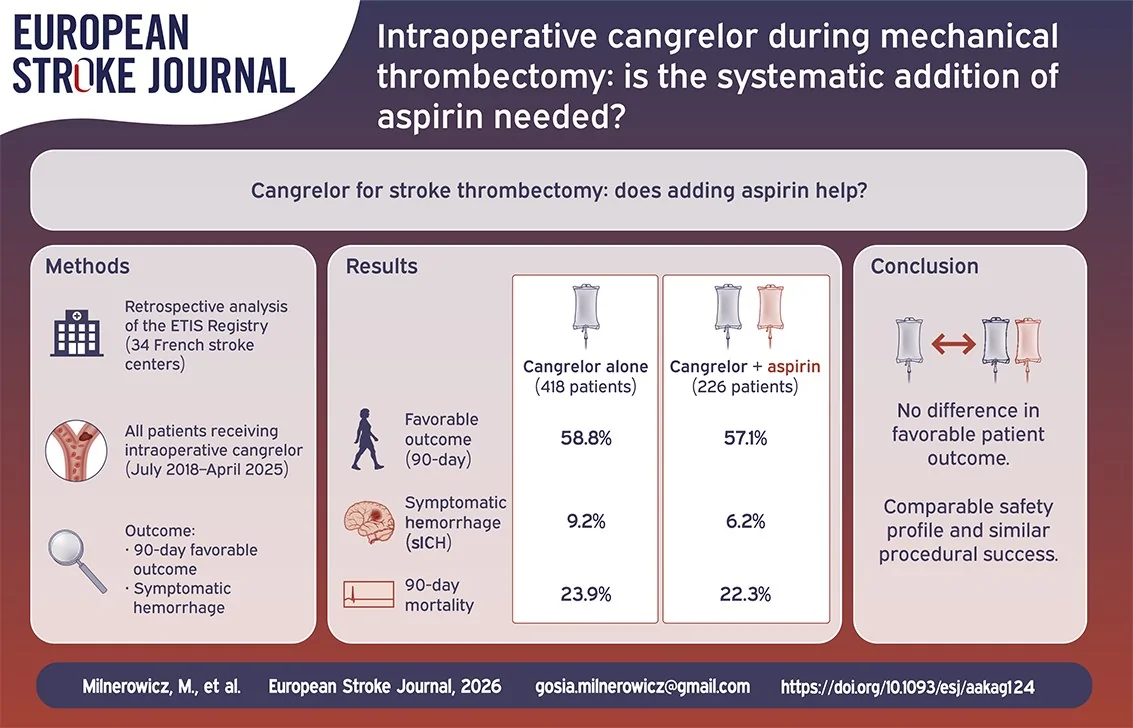

## Introduction

Pivotal randomised controlled trials established mechanical thrombectomy (MT) as the standard treatment for large-vessel occlusion stroke (LVOS). Yet, nearly 15% of procedures still fail to achieve successful recanalisation.^1^ Specific clinical scenarios necessitate additional measures to restore or preserve vessel patency beyond thrombectomy alone. These include tandem occlusions, representing about 15% of all LVOS, intracranial atherosclerotic disease (ICAD), which accounts for 10%–30% of LVOS depending on ethnicity, and selected refractory intracranial occlusions requiring rescue angioplasty or stenting after unsuccessful thrombectomy.^2^^,^^3^ Together, these situations often require acute antiplatelet therapy (APT) combined with endovascular rescue techniques to optimise reperfusion and maintain early vessel patency.

A growing body of evidence supports the role of APT in improving reperfusion quality, reducing early stent thrombosis and ultimately enhancing functional outcomes when applied in specific clinical circumstances.^3–5^ Despite its central role, no well-established or uniform antiplatelet protocol exists for either elective or emergent neuroendovascular procedures, and substantial heterogeneity in APT administration persists among centres performing MT.^6^^,^^7^

Cangrelor has recently emerged as a promising intravenous option in neurointervention due to its rapid onset of action, quick reversibility and favourable safety profile, attributes particularly relevant in the setting of acute ischaemic stroke (AIS).^8–10^ However, as with other potent intravenous antiplatelet agents used during MT, its use is supported predominantly by observational data, and no randomised evidence has formally established cangrelor—or any other agent—as the preferred periprocedural antiplatelet strategy in this setting to date. In recent years, cangrelor has shifted from its historical role as a second-line therapy—typically reserved for situations in which aspirin alone was deemed insufficient or unreliable— to an agent increasingly used as first-line antiplatelet therapy or monotherapy.^11–13^ This evolving practice raises the question of whether concomitant aspirin provides additional benefit when cangrelor is administered during MT. In elective or non-acute neurointerventional procedures requiring intra- or extracranial stent placement, patients traditionally receive dual antiplatelet therapy, raising concerns as to whether cangrelor alone provides sufficient protection against early reocclusion or stent thrombosis in the acute setting. Although several observational studies have suggested a favourable safety profile for cangrelor, data specifically addressing its use with or without coincident intravenous aspirin remain limited.

Therefore, this study aimed to evaluate whether any safety or efficacy signal could be identified with the addition of intravenous aspirin to cangrelor in patients with LVOS undergoing MT.

## Methods

### Study population

We performed a retrospective analysis of patients enrolled in the prospective, multicentre Endovascular Treatment of Ischemic Stroke (ETIS) Registry between July 2018 and April 2025 (NCT03776877). The registry involves 34 comprehensive stroke centres across France and systematically includes all consecutive patients undergoing MT for AIS. Data acquisition and processing were approved by local ethics committees and conducted in accordance with the recommendations of the Comité Consultatif sur le Traitement de l’Information en matière de Recherche dans le domaine de la Santé. All consecutive patients with AIS enrolled in the ETIS registry who underwent MT with intraoperative cangrelor administration were included in the present analysis, regardless of the indication for intravenous APT—whether for a refractory intracranial occlusion, underlying intracranial or cervical artery disease, or procedures involving angioplasty or rescue stent placement. Patients were eligible for inclusion irrespective of concomitant intravenous aspirin administration.

### Treatment

The decision to perform MT followed current guideline-based criteria, taking into account clinical presentation, imaging findings, patient comorbidities and time from symptom onset. When appropriate and in the absence of contraindications, intravenous thrombolysis (alteplase or tenecteplase) was administered. Choices regarding anaesthesia and the specific endovascular technique were left to the operator’s judgement according to local practice, patient status and occlusion characteristics.

Decisions regarding adjunctive pharmacologic or mechanical treatments were made individually. Intravenous APT with cangrelor was considered in situations such as persistent or early recurrent intracranial occlusion despite standard MT manoeuvres, identification of arterial wall pathology suggesting a high risk of reocclusion, or cervical artery disease requiring emergent angioplasty or stenting. These decisions were taken locally through multidisciplinary discussion, considering prior antithrombotic exposure, patient-specific risk factors and angiographic findings.

Cangrelor was delivered according to standard dosing recommendations and local protocols, consisting of an intravenous bolus followed by continuous infusion. Adjunctive intravenous aspirin was given at the operator’s discretion. The duration of intravenous cangrelor administration and the subsequent transition to oral agents were determined according to each centre’s practice and clinical evolution.

### Collected data and outcomes

Clinical, imaging, temporal and angiographic data were prospectively recorded at each site by senior interventional neuroradiologists and neurologists. Functional outcome at 90 days was assessed using the modified Rankin Scale (mRS), either during an in-person consultation with a vascular neurologist or through a structured telephone interview conducted by certified study personnel.

The primary endpoint was a favourable functional outcome, defined as a 90-day mRS score of 0–2 or equal to pre-stroke mRS. Secondary endpoints included excellent functional outcome (mRS 0–1), early neurological improvement defined as an improvement in the National Institutes of Health Stroke Scale (NIHSS) score of at least 4 points within the first 24 h, Alberta Stroke Program Early CT Score (ASPECTS) and NIHSS shift at 24 h and angiographic efficacy, assessed through final modified Thrombolysis in Cerebral Infarction (mTICI), and day-1 patency of the intra- and extracranial target arteries. Day-1 arterial patency was assessed according to each participating centre’s routine imaging protocol using either magnetic resonance imaging (MRI), including time-of-flight and/or contrast-enhanced magnetic resonance angiography or computed tomography angiography (CTA). Safety endpoints comprised intracranial haemorrhage (ICH) and 90-day mortality.

Recanalisation was classified according to the mTICI scale, with successful reperfusion defined as grades 2b–3 and complete reperfusion as grade 3. Intracranial haemorrhage was defined and categorised according to the European Cooperative Acute Stroke Study II (ECASS II) classification. We documented any ICH, parenchymal haematoma and symptomatic ICH (sICH), the latter defined as ICH associated with neurological deterioration (NIHSS score increase ≥4 points or death) within the first 24 h.

### Statistical analysis

Quantitative variables are expressed as means (standard deviation, SD) in the case of normal distribution or medians (interquartile range) otherwise. Categorical variables are expressed as numbers (percentage). Normality of distributions was assessed using histograms and the Shapiro–Wilk test. Patient’s and treatment characteristics were described according the study groups (cangrelor + aspirin combination vs cangrelor alone) and the magnitude of the between-group differences was assessed by calculating the standardised differences. A standardised difference >10% in absolute was interpreted as a meaningful difference.

Main angiographic and clinical outcomes were compared between the two groups before and after taking account the potential confounding factors by using propensity-score (PS) overlap weighting (PSOW) method.^14^ The PS was estimated using a non-parsimonious multivariable logistic regression model, with the study group as the dependent variable and the pre-specified confounders (see Table 1) as covariates.

**Table 1:** Patient’s characteristics according to use of aspirin.

	**Use of aspirin**		
**Characteristics**	**No (*n* = 418)**	**Yes (*n* = 226)**	**ASD, %**
**Age, years, mean ± SD** ^**a**^	66.9 ± 13.0	64.6 ± 13.2	17.6
**Men** ^**a**^	148/418 (35.4)	65/225 (28.9)	14.0
**Mothership admission** ^**a**^	182/413 (44.1)	98/226 (43.4)	1.4
**Unknown onset time** ^**a**^	140/413 (33.9)	73/223 (32.7)	2.5
**Medical history**			
**Hypertension**^**a**^	246/408 (60.3)	121/220 (55.0)	10.7
**Hypercholesterolaemia**^**a**^	141/404 (34.9)	48/212 (22.6)	27.3
**Diabetes mellitus**^**a**^	101/404 (25.0)	40/219 (18.3)	16.4
**Current smoking**^**a**^	118/380 (31.1)	62/214 (29.0)	4.5
**History of previous stroke**^**a**^	63/401 (15.7)	22/217 (10.1)	16.7
**Atrial fibrillation**^**a**^			
**Coronary disease**	32/394 (8.1)	15/214 (7.0)	4.2
**Pre-stroke antithrombotic treatment**^**a**^	133/397 (33.5)	50/213 (23.5)	22.3
**Admission systolic blood pressure, mean ± SD** ^**a**^	152 ± 28	154 ± 28	8.4
**Admission NIHSS score, median (IQR)** ^**a**^	13 (8–18)	12 (7–18)	4.0
**Baseline ASPECTS, median (IQR)** ^**a**^	8 (7–9)	8 (7–9)	0.6
**Pre-stroke mRS >1** ^**a**^	80/360 (22.2)	40/215 (18.6)	9.0
**Site of intracranial occlusion** ^**a**^			
**ICA**	28/414 (6.8)	14/226 (6.2)	44.7
**M1**	134/414 (32.4)	39/226 (17.3)	
**M2**	28/414 (6.8)	8/226 (3.5)	
**Tandem**	125/414 (30.2)	97/226 (42.9)	
**Vertebro basilar**	57/414 (13.8)	49/226 (21.7)	
**Extracranial ICA**	33/414 (8.0)	17/226 (7.5)	
**Other/multiple**	9/414 (2.2)	2/226 (0.9)	
**Intravenous thrombolysis** ^**a**^	154/411 (37.5)	112/225 (49.8)	25.0
**Stroke aetiology**			
**Dissection**	20/348 (5.7)	27/205 (13.2)	36.3
**Cardioembolic**	42/348 (12.1)	16/205 (7.8)	
**Atheroma**	143/348 (41.1)	103/205 (50.2)	
**Others/multiple/indetermined**	143/348 (41.1)	59/205 (28.8)	
**Onset to imaging, min, median (IQR)**	136.5 (98–218)	140 (85–220)	3.4
**Onset to groin puncture, min, median (IQR)** ^**a**^	296 (222–420)	301 (226–410)	3.5
**Procedural characteristics**			
**General anaesthesia**^**a**^	3120 (23.6)	162 (37.4)	30.5
**First line MT strategy**			
**Stentretriever**	7/397 (1.8)	7/203 (3.4)	24.9
**Aspiration**	191/397 (48.1)	111/203 (54.7)	
**Stentretriever + aspiration**	168/397 (42.3)	79/203 (38.9)	
**Others**	31/397 (7.8)	6/203 (3.0)	
**Number of passes**	2 (1 to 4)	2 (1 to 3)	6.6
**Adjuvant treatment**			
**Stenting**	46/301 (15.3)	48/194 (24.7)	25.2
**Angioplasty**	47/301 (15.6)	27/194 (13.9)	
**Stenting + angioplasty**	207/301 (68.8)	119/194 (61.3)	
**Others**	1/301 (0.3)	0/194 (0.0)	

Values are expressed as number (%) unless otherwise indicated. ^a^Included in propensity score. Abbreviations: ASD = absolute standardized difference; ASPECTS = Alberta Stroke Program Early CT Score; ICA = internal carotid artery; IQR = interquartile range; IVT = intravenous thrombolysis; mRS = modified Rankin Scale; MT = mechanical thrombectomy; NIHSS = National Institutes of Health Stroke Scale; SD = standard deviation.

In the unadjusted cohort, comparisons between study groups were done using mixed logistic regression models considering centre as random effect for binary outcomes, and using a linear mixed regression model including centre as random effect and admission scores (NIHSS or ASPECTS) as covariates fixed effects for change in NIHSS and ASPECT scores from baseline to 24 h. Odds ratios and baseline-adjusted mean difference for cangrelor + aspirin combination versus cangrelor alone were, respectively, reported as effect size with theirs 95% confidence intervals (CIs). In the PSOW cohort, the effect sizes were estimated using a weighted mixed logistic or linear regression models including centre as random effect and PS overlap weights, with robust standard errors to account for weighting. To avoid case-deletion in analyses due to missing data in prespecified confounders and outcomes, effect sizes were estimated after handling missing data values by multiple imputation (m = 10 imputations) using a fully conditional specification. Imputation procedure was performed under the missing at random assumption using all variables listed in Table 1 and outcomes with a predictive mean matching method for quantitative variables and multinomial or binary logistic regression model for categorical variables. In each imputed dataset, we calculated the propensity score, the unadjusted and PSOW-adjusted effect sizes, which were later combined by using Rubin’s rules.

All patients were included in the primary analysis. A sensitivity analysis excluding patients receiving antiplatelet therapy prior to the qualifying ischaemic stroke was subsequently performed. Clinical and angiographic outcomes were reassessed using the same methodological approach as in the primary analysis, with adjustment for potential confounders. Statistical testing was conducted at the two-tailed α-level of .05. Data were analysed using the SAS software version 9·4 (SAS Institute, Cary, NC, USA).

## Results

During the study period, 644 patients met the inclusion criteria, with 418 receiving cangrelor alone and 226 receiving cangrelor + aspirin combination. Overall, the patient’s characteristics at admission and treatment characteristics between the groups were presented before (Table S1) and after (Table 1) handling missing values by multiple imputation. Patients receiving the cangrelor + aspirin combination were younger (mean ± SD, 64.6 ± 13.2 vs 66.9 ± 13.0), more frequently women (71.1% vs 64.5%), had lower rates of hypercholesterolaemia (22.6% vs 34.9%), diabetes (18.3% vs 25.0%) and previous stroke (10.1% vs 15.7%), and were less frequently on prior antithrombotic treatment (23.5% vs 33.5%).

Regarding procedural characteristics, patients in the cangrel-or + aspirin group were more frequently treated under general anaesthesia (37.4% vs 23.6%) and more often underwent aspiration alone as the first-line MT strategy (54.7% vs 48.1%). Adjuvant stenting was performed more frequently in patients receiving cangrelor + aspirin than in those treated with cangrelor alone (24.7% vs 15.3%). At admission, NIHSS and ASPECT scores were comparable, and large artery atherosclerosis was the most common aetiology in both groups (50.2% vs 41.1%). Tandem lesions were the most frequent occlusion site (42.9% vs 30.2%), followed by M1 (17.3% vs 32.4%) and basilar or vertebral arteries (21.7% vs 13.8%).

### Clinical and angiographic outcomes

In the PSOW-weighted cohort, the primary endpoint did not differ significantly between groups: favourable 3-month outcomes were observed in 57.1% of patients receiving cangrelor + aspirin and 58.8% of those treated with cangrelor alone (aOR = 0.93; 95% CI, 0.67–1.30; *P* = 0.69). Rates of excellent functional outcome at 90 days and early neurological improvement were also comparable (Table 2). Likewise, 24-h changes in NIHSS and ASPECTS showed no significant between-group differences, with adjusted mean differences of 0.7 (95% CI, –1.0 to 2.4) and –0.1 (95% CI, –0.6 to 0.4), respectively.

**Table 2:** Comparisons of clinical and procedural outcomes according to aspirin use after propensity score overlap weighting.

	**Use of aspirin**			
	**No (*n* = 418)**	**Yes (*n* = 226)**	**Effect size (95% CI)**	***P* value**
**Angiographic efficacy outcomes (%)**				
**Final mTICI 2b-2c-3**	88.8	89.4	1.07 (0.71 to 1.61)	.76
**Final mTICI 3**	38.3	45.1	1.32 (0.86 to 2.03)	.21
**Intracranial patency at 24 h**	83.5	87.5	1.39 (0.81 to 2.38)	.23
**Extracranial artery patency at 24 h**^**a**^	92.6	96.0	1.94 (0.72 to 5.23)	.19
**Clinical efficacy outcomes (%)**				
**Favourable outcome**^**b**^	58.8	57.1	0.93 (0.67 to 1.30)	.69
**Excellent outcome**^**c**^	41.1	42.6	1.07 (0.67 to 1.71)	.79
**24-h ASPECTS shift, mean (SD)**	−0.7 (3.0)	−0.8 (3.6)	−0.1 (−0.6 to 0.4)^**d**^	.57
**24-h NIHSS shift, mean (SD)**	−1.2 (12.7)	−0.7 (16.4)	0.7 (−1.0 to 2.4)^**d**^	.44
**Early neurological improvement (%)**	27.6	23.2	0.79 (0.56 to 1.12)	.18
**Safety outcomes**				
**Intracranial haemorrhagic complications (%)**				
**Any ICH**	42.8	39.1	0.86 (0.67 to 1.09)	.21
**sICH**	9.2	6.2	0.66 (0.38 to 1.13)	.20
**PH**	10.0	10.7	1.07 (0.67 to 1.72)	0.76
**90-day mortality (%)**	23.9	22.3	0.91 (0.57 to 1.45)	.70
**Procedural complication (%)**	19.6	18.0	0.90 (0.53 to 1.53)	.71

^a^Values are estimated in propensity-score overlap weighting cohort unless otherwise indicated. aamong patients with occlusion in extracranial arteries (isolated or in tandem with intracranial arteries).

^b^Pre-specified primary outcome defined as a 90-day mRS of 0–2, or equal to pre-stroke mRS.

^c^Defined as a 90-day mRS of 0–1, or equal to pre-stroke mRS.

^d^Baseline-adjusted mean difference. Descriptive parameters and effect sizes (odds ratio or mean difference) were calculated after handling missing values for variables included in the propensity score using multiple imputations.

Abbreviations: ASPECTS = Alberta Stroke Program Early CT Score; CI = confidence interval; ICH = intracerebral haemorrhage; IQR = interquartile range; mRS = modified Rankin Scale; mTICI = modified Thrombolysis in Cerebral Infarction; NIHSS = National Institutes of Health Stroke Scale; PH = parenchymal haemorrhage of type 1 and 2; SD = standard deviation; sICH = symptomatic intracerebral haemorrhage.

In terms of angiographic outcomes, successful reperfusion (final mTICI ≥2b) was achieved in 89.4% of patients in the cangrelor + aspirin group and 88.8% in the cangrelor-alone group (aOR = 1.07; 95% CI, 0.71–1.61; *P* = 0.76). Rates of complete reperfusion (mTICI 3) were similarly comparable (aOR = 1.32; 95% CI, 0.71–1.61; *P* = 0.76). Day-1 intracranial and extracranial arterial patency also showed no notable differences (Table 2).

Regarding safety outcomes, no significant differences were observed in 90-day mortality (22.3% vs 23.9%, aOR = 0.91; 95% CI, 0.57–1.45), symptomatic ICH (6.2% vs 9.2%, aOR = 0.66; 95% CI, 0.38–1.13) or overall ICH (39.1% vs 42.8%, aOR = 0.86; 95% CI, 0.67–1.09). Similar results were found in unadjusted cohort (Table S1).

In the sensitivity analysis including only patients without pre-stroke antiplatelet therapy, results were consistent with those of the overall population, except for sICH, which occurred more frequently in the cangrelor-alone group compared with the cangrelor + aspirin group (8.9% vs 4.6%; aOR = 0.49; 95% CI, 0.26–0.93) (Table 3).

**Table 3:** Comparisons of clinical and procedural outcomes according to aspirin use after propensity-score overlap weighting in patients without pre-stroke antiplatelet therapy.

	**Use of aspirin**			
	**No (*n* = 328)**	**Yes (*n* = 195)**	**Effect size (95% CI)**	***P* value**
**Angiographic efficacy outcomes (%)**				
**Final mTICI 2b-2c-3**	89.3	90.5	1.14 (0.62 to 2.10)	.67
**Final mTICI 3**	39.9	46.8	1.33 (0.84 to 2.09)	.22
**Intracranial patency at 24 h**	83.9	84.5	1.05 (0.62 to 1.75)	.86
**Extracranial artery patency at 24 h**^**a**^	93.3	96.5	2.01 (0.59 to 6.79)	.26
**Clinical efficacy outcomes (%)**				
**Favourable outcome**^**b**^	61.5	58.5	0.88 (0.60 to 1.30)	.52
**Excellent outcome**^**c**^	44.3	45.0	1.03 (0.58 to 1.82)	.92
**24-h ASPECTS shift, mean (SD)**	−0.6 (3.0)	−0.7 (3.4)	−0.1 (−0.6 to 0.3)^**d**^	.61
**24-h NIHSS shift, mean (SD)**	−1.3 (12.9)	−1.1 (14.8)	0.7 (−1.3 to 2.8)^**d**^	.50
**Early neurological improvement**	26.8%	22.3%	0.79 (0.51 to 1.20)	.26
**Safety outcomes**				
**Intracranial haemorrhagic complications (%)**				
**Any ICH**	43.3	37.6	0.79 (0.60 to 1.04)	.087
**sICH**	8.9	4.6	0.49 (0.26 to 0.93)	.028
**PH**	9.4	9.8	1.05 (0.63 to 1.73)	.86
**90-day mortality**	22.0	21.1	0.94 (0.56 to 1.60)	.83
**Procedural complication**	19.5	15.9	0.78 (0.47 to 1.30)	.34

^a^Values are estimated in propensity-score overlap weighting cohort unless otherwise indicated. aamong patients with occlusion in extracranial arteries (isolated or in tandem with intracranial arteries).

^b^Pre-specified primary outcome defined as a 90-day mRS of 0–2, or equal to pre-stroke mRS.

^c^Defined as a 90-day mRS of 0–1, or equal to pre-stroke mRS.

^d^Baseline-adjusted mean difference. Descriptive parameters and effect sizes (odds ratio or mean difference) were calculated after handling missing values for variables included in the propensity score using multiple imputations.

Abbreviations: ASPECTS = Alberta Stroke Program Early CT Score; CI = confidence interval; ICH = intracerebral haemorrhage; IQR = interquartile range; mRS = modified Rankin Scale; mTICI = modified Thrombolysis in Cerebral Infarction; NIHSS = National Institutes of Health Stroke Scale; PH = parenchymal haemorrhage of type 1 and 2; SD = standard deviation; sICH = symptomatic intracerebral haemorrhage.

## Discussion

In this large multicentre cohort, clinical, angiographic and safety outcomes were not significantly different between patients treated with intraoperative cangrelor alone and those receiving cangrelor in combination with intravenous aspirin. Functional outcome, early neurological improvement, reperfusion success, day-1 intra- and extracranial patency, intracranial haemorrhage and 90-day mortality did not significantly differ between groups. Accordingly, this study addresses the specific question of whether the addition of intravenous aspirin to cangrelor is associated with a safety or efficacy signal, rather than the broader question of the optimal periprocedural antiplatelet strategy during MT. These findings should be interpreted in the context of a population in whom cangrelor had already been selected as the periprocedural antiplatelet strategy. The absence of significant differences in this observational cohort should therefore be interpreted cautiously, as it neither demonstrates equivalence between the two approaches nor formally rules out a potential benefit of adjunctive aspirin.

To date, neither cangrelor nor glycoprotein (GP) IIb/IIIa inhibitors have been established as a reference periprocedural antiplatelet strategy by randomised trials, and their use during MT remains largely guided by observational evidence and local practice. In this context, comparative data on currently used treatment combinations remain clinically relevant and may help inform future prospective studies.

The clinical interest in evaluating this combination emerges from the challenge of balancing antithrombotic protection and haemorrhagic risk in a context of AIS inherently prone to intracranial bleeding. Both initial recanalisation and early vessel patency are essential determinants of neurological recovery. In ICAD-related or otherwise refractory intracranial occlusions, achieving recanalisation remains a pivotal determinant of recovery, bringing clinical outcomes closer to those observed in standard MT populations.^15^^,^^16^ Durable arterial patency after MT is an important prognostic marker in both intracranial and extracranial occlusions. Delayed reocclusion or impaired arterial patency has consistently been associated with early neurological deterioration, increased mortality and poorer functional outcomes.^17^ This association has also been demonstrated in specific clinical settings, including extracranial internal carotid artery lesions, where day-1 carotid patency independently predicts favourable 3-month functional outcome irrespective of intracranial recanalisation success,^4^ and rescue intracranial stenting after failed first-line MT, where persistent intracranial stent patency is likewise associated with favourable clinical outcome.^18^

Previous studies have shown that aggressive APT—either with GP IIb/IIIa inhibitors or cangrelor—can improve early patency without significantly increasing symptomatic ICH.^5^^,^^13^ Similar benefits have also been observed with combined antiplatelet regimens, whether administered orally or intravenously alongside aspirin.^5^^,^^19^ Nonetheless, aspirin monotherapy has repeatedly been associated with higher early stent occlusion rates, and thrombosis occurring within the first 24 h has emerged as the key determinant of prognosis, supporting the importance of effective periprocedural antiplatelet coverage.^4^^,^^19^

Yet, the potential benefit of adding aspirin must be weighed against concerns regarding early haemorrhagic risk. The MR CLEAN-MED trial demonstrated increased sICH with intravenous aspirin during MT without functional benefit, raising questions about its systematic intraoperative use.^20^ Reports of major haemorrhage with triple antiplatelet combinations involving aspirin, clopidogrel and GP IIb/IIIa inhibitors—particularly abciximab—underline the need for caution.^11^^,^^21^ In contrast, some studies describe a more favourable safety profile: Pop et al. reported that an aggressive regimen combining aspirin, clopidogrel and tirofiban improved patency and intermediate outcomes without increasing mortality or ICH.^19^ Overall, the evidence remains heterogeneous and at times conflicting, and the majority of available data pertains to GP IIb/IIIa inhibitors rather than cangrelor.

Evidence regarding the concomitant use of aspirin and cangrelor remains limited, with most available data derived from small series, and no dedicated aspirin-based subgroup analyses in larger cohorts or meta-analyses despite its widespread use in clinical practice.^8^^,^^9^ Such variability reflects persistent local protocols initiating aspirin first and adding cangrelor only after suboptimal angiographic response, which contributes to group heterogeneity.

In our study, adjunctive aspirin was not associated with adverse clinical or safety outcomes in the overall population. Sensitivity analyses restricted to patients without pre-stroke antiplatelet therapy yielded broadly consistent results for most outcomes. A lower rate of sICH was observed in the cangrelor + aspirin group compared with cangrelor alone; however, this finding was unexpected and its interpretation remains uncertain, particularly given the amount of missing data for this outcome. This isolated signal should therefore be interpreted cautiously and requires confirmation in further studies.

Recent publications have further explored cangrelor monotherapy in comparison with GP IIb/IIIa inhibitors, and in one study also relative to aspirin.^10^^,^^13^ Pop et al. found that cangrelor improved carotid stent patency compared with aspirin alone, although functional outcomes were not different, and cangrelor appeared to yield worse 90-day outcomes than GP IIb/IIIa inhibitors. This trend, however, was not confirmed in the meta-analysis by Gonçalves et al., who reported comparable functional outcomes and similar sICH rates between both drug classes, with improved reperfusion in the cangrelor group.

In our cohort, no significant differences were detected in recanalisation, 24-h arterial patency, recanalisation success, overall clinical outcomes or safety measures between patients receiving cangrelor alone and those receiving adjunctive aspirin. However, given the observational design, clinical heterogeneity, and potential for residual confounding, a differential benefit of adjunctive aspirin cannot be formally excluded.

Beyond the choice of single or combined antiplatelet therapy, optimisation of cangrelor dosing may represent another important aspect of periprocedural management in acute stroke. The currently recommended cardiovascular regimen was developed for patients undergoing percutaneous coronary intervention and may not be directly transferable to the neuroendovascular setting. Reduced-dose regimens have therefore gained interest as a strategy to maintain platelet inhibition while potentially reducing haemorrhagic risk and treatment costs.^22^ This evolution in practice was also observed among centres participating in our study, with some operators progressively transitioning from the full cardiovascular dose to half-dose or lower-dose regimens during the study period. Although this contributed to heterogeneity in cangrelor administration protocols and might be a potential source of bias in the current study, it also reflects an emerging shift in clinical practice and highlights dose optimisation as a particularly relevant area for future investigation.

One practical consideration, however, is that reliance on cangrelor alone requires strict continuity of infusion, as any unintentional interruption may transiently leave patients without effective platelet inhibition, potentially exposing them to a reocclusion risk.

This study had several limitations inherent to its retrospective, non-randomised design. Importantly, the decision to administer adjunctive intravenous aspirin was not randomised and remained at the discretion of the treating team, introducing potential confounding by indication. The choice to add aspirin may have been influenced by clinical, anatomical and procedural factors, including tandem lesions, underlying intracranial atherosclerotic disease, perceived thrombotic or reocclusion risk, stenting strategy, procedural complexity, and local or operator preference. The observed baseline differences between groups are consistent with such treatment selection and suggest that adjunctive aspirin may have been preferentially used in patients perceived to be at greater thrombotic risk. Although PSOW can account for measured covariates, it cannot fully address determinants of treatment choice that were not captured in the registry. In addition, the study was not designed or powered to demonstrate equivalence or non-inferiority between treatment strategies. Therefore, the absence of statistically significant differences should not be interpreted as evidence that adjunctive aspirin provides no additional benefit, but rather as an inability to demonstrate a measurable difference between the two strategies within this observational cohort. A potential benefit of adjunctive aspirin therefore cannot be excluded.

The presence of missing values in several covariates and outcomes also introduced potential bias, although established multiple imputation procedures were applied.

As in other multicentre observational cohorts, treatment protocols were not standardised. Cangrelor dosing and infusion duration varied across centres, and data regarding the timing and choice of oral antiplatelet transition were not available, reflecting the broader absence of harmonised periprocedural antiplatelet protocols in acute ischaemic stroke.

The study population was also clinically heterogeneous, encompassing cervical and intracranial arterial disease of different aetiologies and patients undergoing different rescue strategies, including angioplasty and stenting. These conditions may differ in their underlying thrombotic mechanisms, reocclusion risk and response to antiplatelet therapy. The present study was not designed to reliably assess treatment effects within these individual clinical or procedural subgroups, and a differential effect of adjunctive aspirin in selected settings therefore cannot be excluded.

In particular, approximately one-third of patients in the cangrelor monotherapy group were receiving antiplatelet therapy before stroke onset. Although this imbalance was addressed using PSOW and a dedicated sensitivity analysis restricted to patients without pre-stroke antiplatelet therapy, it should be considered when interpreting the results.

Finally, imaging and angiographic data were assessed locally without centralised review, which may have contributed to inter-centre variability in the evaluation of reperfusion and arterial patency. In addition, the assessment of day-1 arterial patency was based on local imaging protocols, using either MRI or CTA depending on the participating centre, which may have introduced additional variability in outcome assessment.

Despite these limitations, this large multicentre study provides valuable observational data on periprocedural antiplatelet management during MT and may help inform the design of future prospective studies.

## Conclusion

In this large multicentre observational cohort of patients receiving cangrelor during endovascular treatment for LVOS, the addition of intravenous aspirin was not associated with significant differences in clinical, angiographic or safety outcomes. These results provide comparative evidence on two cangrelor-based strategies increasingly encountered in acute neurointerventional practice, although they do not establish equivalence or exclude a potential benefit of adjunctive aspirin. Prospective studies are now needed to identify the clinical settings in which aspirin may provide additional benefit and to refine periprocedural antiplatelet strategies during MT.

## Supplementary Material

Supplementary_material_aakag124

## Abbreviations

aOR, adjusted odds ratio; AIS, acute ischaemic stroke; ASPECTS, Alberta Stroke Program Early Computed Tomography Score; APT, antiplatelet therapy; CI, confidence interval; CTA, computed tomography angiography; ECASS II, European Cooperative Acute Stroke Study II; ETIS, Endovascular Treatment of Ischemic Stroke; GP IIb/IIIa, glycoprotein IIb/IIIa; ICA, internal carotid artery; ICAD, intracranial atherosclerotic disease; ICH, intracranial haemorrhage; IQR, interquartile range; LVOS, large vessel occlusion stroke; MRI, magnetic resonance imaging; mRS, modified Rankin Scale; MT, mechanical thrombectomy; mTICI, modified Thrombolysis in Cerebral Infarction; NIHSS, National Institutes of Health Stroke Scale; PS, propensity score; PSOW, propensity-score overlap weighting; sICH, symptomatic intracranial haemorrhage; SD, standard deviation
