# Supplementary material for: Intraoperative cangrelor during mechanical thrombectomy: is the systematic addition of aspirin needed?
Source: Eur Stroke J. 2026 Oct 19;11(10):aakag124. doi: 10.1093/esj/aakag124 (PMC13679750; doi:10.1093/esj/aakag124)
Supplement: Supplementary file 1 [file supplementary_material_aakag124.zip › Supplementary_material_caption.docx]

**Supplemental Table 1. Comparisons in clinical and procedural outcomes according to use or not of aspirin before Propensity Score overlap weighting.**

|  | Before imputation | |  |  |  | After imputation^4^ | | | |
| --- | --- | --- | --- | --- | --- | --- | --- | --- | --- |
|  | **Use of aspirin** | |  |  |  | **Use of aspirin** | |  |  |
|  | **No**  **(n=418)** | **Yes**  **(n=226)** | **Effect size (95%CI)** | **p-value** |  | **No**  **(n=418)** | **Yes**  **(n=226)** | **Effect size (95%CI)** | **p-value** |
| Angiographic efficacy outcomes |  |  |  |  |  |  |  |  |  |
| Final mTICI 2b-2c-3 | 336/381 (88.2) | 191/212 (90.1) | 1.24 (0.71 to 2.18) | 0.45 |  | 367 (87.9) | 204 (90.3) | 1.29 (0.82 to 2.02) | 0.27 |
| Final mTICI 3 | 139/381 (36.5) | 93/212 (43.9) | 1.37 (0.93 to 2.04) | 0.11 |  | 154 (36.7) | 101 (44.6) | 1.39 (0.92 to 2.09) | 0.12 |
| Intracranial patency at 24 hours | 261/309 (84.5) | 130/147 (88.4) | 1.41 (0.78 to 2.55) | 0.26 |  | 261 (84.5) | 130 (88.4) | 0.93 (0.67 to 1.30) | 0.67 |
| Extracranial artery patency at 24 hours | 95/101 (94.1) | 55/57 (96.5) | 1.94 (0.39 to 9.82) | 0.42 |  | 137/150 (91.6) | 91/96 (95.0) | 1.75 (0.70 to 4.35) | 0.23 |
| Clinical efficacy outcomes |  |  |  |  |  |  |  |  |  |
| Favorable outcome^1^ | 211/348 (60.6) | 118/198 (59.6) | 0.82 (0.55 to 1.23) | 0.34 |  | 240 (57.4) | 133 (58.6) | 1.05 (0.75 to 1.48) | 0.78 |
| Excellent outcome^2^ | 149/339 (44.0) | 92/197 (46.7) | 0.87 (0.58 to 1.32) | 0.51 |  | 167 (39.9) | 100 (44.2) | 1.19 (0.72 to 1.97) | 0.50 |
| 24-hour ASPECTS shift, mean (SD) | -0.6 (2.4) | -0.7 (1.9) | -0.24 (-0.71 to 0.22)^3^ | 0.30 |  | -0.7 (2.5) | -0.8 (2.1) | -0.1 (-0.6 to 0.4) ^3^ | 0.68 |
| 24-hour NIHSS shift, mean (SD) | -1.9 (9.6) | -1.7 (9.1) | 1.31 (-0.52 to 3.16)^3^ | 0.16 |  | -1.1 (10.2) | -1.6 (9.8) | 0.5 (-2.2 to 1.4) ^3^ | 0.65 |
| Early neurological improvement | 95/316 (30.1) | 43/172 (25.0) | 0.68 (0.42 to 1.11) | 0.13 |  | 114 (27.2) | 58 (25.7) | 0.93 (0.68 to 1.26) | 0.62 |
| Safety outcomes |  |  |  |  |  |  |  |  |  |
| Intracranial hemorrhagic complications |  |  |  |  |  |  |  |  |  |
| Any ICH | 152/418 (36.4) | 80/226 (35.4) | 1.13 (0.74 to 1.73) | 0.57 |  | 178 (42.5) | 91 (40.3) | 0.91 (0.66 to 1.27) | 0.60 |
| sICH | 34/418 (8.1) | 14/226 (6.2) | 0.77 (0.40 to 1.50) | 0.45 |  | 38 (9.1) | 15 (6.8) | 0.73 (0.43 to 1.24) | 0.24 |
| PH | 31/418 (7.4) | 24/226 (10.6) | 1.36 (0.73 to 2.51) | 0.33 |  | 41 (9.9) | 27 (11.9) | 1.23 (0.77 to 1.95) | 0.39 |
| 90-day mortality | 93/373 (24.9) | 47/203 (23.2) | 1.04 (0.65 to 1.65) | 0.88 |  | 100 (23.9) | 50 (22.4) | 0.92 (0.55 to 1.54) | 0.68 |
| Procedural complication | 83/415 (20.0) | 35/220 (15.9) | 0.70 (0.43 to 1.14) | 0.15 |  | 84 (20.0) | 36 (15.9) | 0.76 (0.48 to 1.20) | 0.24 |

^1^ Pre-specified primary outcome defined as a 90-day mRS score of 0 to 2, or equal to pre-stroke mRS score. ^2^ Defined as a 90-day mRS score of 0 to 19, or equal to pre-stroke mRS score. ^3^ Baseline-adjusted mean difference. ^4^Descriptive parameters and effect sizes (odds ratio or mean difference) were calculated after handling missing values for variables included in the propensity score using multiple imputations. Abbreviations: ASPECTS=Alberta Stroke Program Early CT Score, CI=confidence intervals, ICH=intracerebral hemorrhage, IQR=interquartile range, NIHSS=National Institutes of Health Stroke Scale, PH=parenchymal hemorrhage of type 1 and 2, sICH=symptomatic intracerebral hemorrhage.
