# Supplementary material for: Intraoperative cangrelor during mechanical thrombectomy: is the systematic addition of aspirin needed?
Source: Eur Stroke J. 2026 Oct 19;11(10):aakag124. doi: 10.1093/esj/aakag124 (PMC13679750; doi:10.1093/esj/aakag124)
Supplement: Supplementary file 1 [file supplementary_material_aakag124.zip › ETIS investigators.docx]

**ETIS investigators**:

Bertrand Lapergue, Adrien Wang, Arturo Consoli, Oguzhan Coskun, Federico Di Maria, Silvia Pizzuto, Alessandro Sgreccia, Charline Benoit, Lucas Gorza, David Weisenburger-Lile, Waliyde Jabeur, Maia Tchikviladze, Serge Evrard, Georges Rodesch, Raphaël Blanc, Michel Obadia, Jean-Philippe Desilles, Michel Piotin, Pierre Seners, Stanislas Smajda, Simon Escalard, Benjamin Maier, Candice Sabben, Hocine Redjem, Mikael Mazhigi, Grace Adwane, François Delvoye, Amira Al Raaisi, William Boisseau, Adnan Altayeb, Erwan Robichon, Igor Raynouard, Cristina Hobeanu, Omer Eker, Tae-Hee Cho, Laurent Derex, Julia Fontaine, Laura Mechtouff, Norbert Nighoghossian, Ong Elodie, Lucie Rascle, Roberto Riva, Françis Turjman, Morgane Laubacher, Mehdi Beyragued, Yves Berthezene, Marc Hermier, Ameli Roxanna, Alexandre Bani-Sadr, Andrea Filip, Matteo Cappucci, Pierre Pernot, Benjamin Daumas Duport, Pierre Louis Alexandre, Hubert Desal, Solene De Gaalon, Benoît Guillon, Cécile Preterre, Guillaume Tessier, Arthur Lionnet, Benjamin Gory, Lisa Humbertjean-Selton, René Anxionnat, Anne-Laure Derelle, Liang Liao, Emmanuelle Schmitt, Sophie Planel, Sébastien Richard, Gioia Mione, Jean-Christophe Lacour, Marian Douarinou, Emilien Micard, Bailiang Chen, Gérard Audibert, Agnès Masson, Alb Lionel, Marine Beaumont, Adriana Tabarna, Marcela Voicu, Grégoire Barthel, Iona Podar, Madalina Brezeanu, Marie Reitter, François Zhu, René Anxionnat, Gaultier Marnat, Jean-Sébastien Liegey, Pierre Briau, Victoire Lyon, Igor Sibon, Xavier Barreau, Jean Papaxanthos, Jerome Berge, Sabrina Debruxelles, Stéphane Olindo, Mathilde Poli, Pauline Renou, Sharmila Sagnier, Thomas Tourdias, Thomas Courret, Malgorzata Milnerowicz, François Rouanet, Benoit Legghe, Aurelie Boyer, Tristan Kerdraon, Lisa Saleille, Laurine Bastardo, Cyril Dargazanli, Vincent Costalat, Federico Cagnazzo, Pierre-Henri Lefevre, Grégory Gascou, Quentin Varnier, François Louis Collemiche, Isabelle Mourand, Caroline Arquizan, Lucas Corti, Adrien Ter Schiphorst, Imad Derraz, Laurent Spelle, Jildaz Caroff, Christian Denier, Vanessa Chalumeau, Cristian Mihalea, Nicolas Legris, Augustin Ozanne, Leon Ikka, Olivier Chassin, Sophie Gallas, Laura Venditti, Mariana Sarov, Jonathan Cortese, Jean-Christophe Ferre, Stephane Vannier, Maria Veronica Lassalle, Jean-Yves Gauvrit, Clément Tracol, Cécile Malrain, Edouard Beaufreton, Quentin Alias, Julien Hissier, Maud Guillen, François Eugene, Julien Boucherit, Cyril Chivot, Audrey Courselle, Sandrine Canaple, Chantal Lamy, Kevin Delaforge, Manuel Fernandez, Jérémie Vial, Quentin Laferte, Xavier Desdoit, Serge Timsit, Aurore Jourdain, Jean-Christophe Gentric, Julien Ognard, Mourad Cheddad El Aouni, Irina Viakhireva, Jordan Coris, François-Mathias Merrien, Denis Marechal, Marie Bruguet, Philippe Goas, Marion Boulanger, Emmanuel Touze, Charlotte Barbier, Romain Schneckenburger, Julien Cogez, Sophie Guettier, Estelle La Porte, Jean Bouchart, Mohammad Ismail, Charbel Mounayer, Aymeric Rouchaud, Suzana Saleme, Géraud Forestier, Sonia Alamowitch, Charlotte Rosso, Flore Baronnet, Sophie Crozier, Anne Leger, Laure Bottin, Sam Ghazanfari, Marion Yger, Stephen Delorme, Aymeric Wittwer, Christine Vassilev, Gaspard Gerschenfeld, Clémence Blanc, Antonio Rainone, Frédéric Clarençon, Kevin Premat, Eimad Shotar, Stéphanie Lenck, Nader Sourour, Julien Allard, Pierre Marie Chiaroni, Olivier Naggara, Guillaume Turc, Wagih Ben Hassen, Denis Trystram, Christine Rodriguez-Regent, Laza-Nomenjanahary Finoana, Hugo Gortais, Ozlem Ozkul-Wermester, Chrysanthi Papagiannaki, Evelyne Massardier, Aude Triquenot, Margaux Lefebvre, Julien Burel, Florian Basille, Adelya Curado, Alain Viguier, Christophe Cognard, Anne Christine Januel, Jean-François Albucher, Lionel Calviere, Jean-Marc Olivot, Nicolas Raposo, Fabrice Bonneville, Guillaume Bellanger, Louis Fontaine, Philippe Tall, Federico Sacchetti, Frédéric Bourdain, Patricia Bernady, Guillaume Ballan, Stephanie Bannier, Emmanuel Ellie, Olivier Flabeau, Julia Potenza, Antoine Soulages, Laurent Lagoarde-Segot, Hélène Cailliez, Louis Veunac, David Higue, Quentin Bourgeois-Beauvais, Anthony Lebras, Sarah Adam, Benoit Pegat, Arnaud Le Guen, François Chedeville, Jérémy Jouan, Rémi Allibert, Alexandre Courtois, Lucile Lescot, Dorothée Videt, Thibaut Lapotre, Johann Sebastian Richter, Bruno Thierry Barroso, Camille Dahan, Alexis Gonnet, Régis Hubrecht, Zoé Lepine, Hélène Castagnet, Raluca Marasescu, Olivier Heck, Pauline Cuisenier, Olivier Detante, Isabelle Favre Wiki, Clémentine Bonaz, Katia Garambois, Loic Legris, Adrian Kastler, Kamel Boubagra, Corentin Berthet, Stéphane Charara, Florent Lepilliet, Jérémie Papassin, François Lun, Carole Henry, Laurence Berthon, Mélanie Roussel, Richard Macrez, Serkan Cakmak, Victor Dumas, Jean Philippe Neau, Nathalie Nasr, Valérie Wolff, Raoul Pop, Véronique Quenardelle, Valérie Lauer, Irène Pierre-Paul, Roxana Gheoca, Leonard Lorant, Solène Moulin, Vi Tuan Hua, Paolo Pagano, Alexandre Doucet, Christophe Gelmini, Pierre-François Manceau, Laurenti Paiusan, Isabelle Serre, Sébastien Soize, Thi Ngoc Phuong Nguyen, Maher Sahnoun, Nathalie Caucheteux, Pablo Lebedinsky, Federico Bolognini, Francis Vuillemet, Anna Ferrier, Abderrahim Zerroug, Ricardo Moreno, Emmanuel Chabert, Elie Lteif, Pauline Paris, Nathalie Bourgois, Marie Raquin, Anne Pasco-Papon, Jean Baptiste Girot, Alderic Lecluse, Sophie Godard, Vincent L'allinec, Alice Corfu, Kevin Janot, Richard Bibi, Marie Gaudron, Arnaud Bretonniere, Mariam Annan, Héloïse Ifergan, Grégoire Boulouis, Marco Pasi, Séverine Debiais, Elisabeth Molinier, Denis Herbreteau, Fouzi Bala, Clémence Hoche, Anthony Wietrich, Valérie Ruche, Karine Lavandier, Hakim Amara, Yannick Bejot, Brivael Lemogne, Frédéric Ricolfi, Laura Baptiste, Pierre Thouant, Gaulthier Duloquin, Pierre-Olivier Comby, Christelle Blanc-Labarre, Simon Amaral, Angélique Bernard, Adrien Chavent, Alan Rhamani, Corinne Chevalier, Guillaume Charbonnier, Louise Bonnet, Nicolas Raybaud, Benjamin Bouamra, Thierry Moulin, Alessandra Biondi, Fernando Pico, Benjamin Dano, Carmen Patcas, Dhouha Chaari, Daniela Stanciu, Duc Long Duong, Intissar Hmida, Marie-Laure Chadenat, Laurent Suissa, Emilie Doche, Jean-François Hak, Anthony Reyre, Xavier Carle, Nadia Laksiri, Ophélie Osman, Roxane Peres, Emmanuelle Robinet-Borgmano, Cécile Dulau, Basile Kerleroux, Caroline Rey, Hervé Brunel, Philippe Dory-Lautrec, Jean-François Hak, Pierre Lehmann, Sivadji Vingadalassalom, Basile Kerleroux, Jérôme Berge, Federico Sacchetti, Fabrice Bing, Mathieu Bonnerot, Isabelle Berger, Nathalie Morel, Gilles Rodier, Wilfried Vadot, Barbara Casolla
